# Experimental Simulation of Hydrocarbon Expulsion in Semi-open Systems from Variable Organic Richness Source Rocks

**DOI:** 10.1021/acsomega.1c01800

**Published:** 2021-05-24

**Authors:** Lianhua Hou, Haiping Huang, Chun Yang, Weijiao Ma

**Affiliations:** † Research Institute of Petroleum Exploration & Development, PetroChina, Beijing 100083, P. R. China; ‡ School of Geosciences, Yangtze University, Wuhan, Hubei 430100, P. R. China; § Department of Geoscience, University of Calgary, 2500 University Drive NW, Calgary, Alberta T2N 1N4, Canada; ∥ School of Geosciences, China University of Petroleum, Qingdao, Shandong 266580, P. R. China

## Abstract

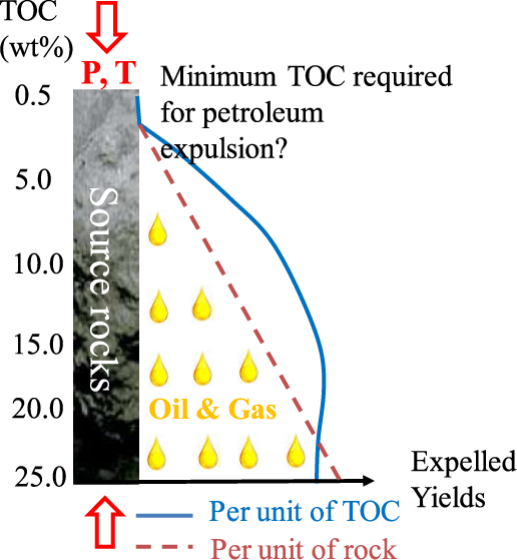

To better understand oil and gas generation and expulsion mechanisms and their controlling factors, two-stage heating program (20 and 5 °C/d) at 11 target temperatures (250–580 °C) have been performed in a semi-open reactor on nine immature lacustrine shale samples from the Triassic Yanchang Formation in Ordos Basin, NW China, with total organic carbon (TOC) contents ranging from 0.5% to 30.0%. The cumulative expelled oil and gas were quantified and correlated with the measured vitrinite reflectance (%*R*_o_) and residual TOC. The amount of expelled oil increases substantially with increasing maturity in the *R*_o_ range of 0.5–1.25% and ends at *R*_o_ of >1.45%, while the volume of expelled gas increases markedly with maturity when *R*_o_ is >1.0%. Organic richness exerts primary control on the expulsion yields, which increase linearly with increasing original TOC (TOC_o_) per unit weight of rock, whereas the increment decreases with TOC_o_ per unit weight of TOC, once the TOC_o_ content is above 5%. Marked TOC reduction occurs in the *R*_o_ of 0.5–1.1% due to oil generation and expulsion, but the trend is reversed in the higher maturity range possibly caused by the simultaneous decomposition of minerals. Numerical correlations among heating temperatures, %*R*_o_, TOC content, and expelled oil and gas yields have been constructed, and the minimum TOC contents for effective oil and gas source rocks have been inferred. The lowest TOC contents of 0.5% and 0.48% are required for oil and gas expulsion in the oil and gas generation window, corresponding to the TOC_o_ of 0.91% and 0.76%, respectively. The minimum TOC content for effective gas source rocks decreases slightly with increasing maturity; however, a much higher TOC cutoff is required for lower maturity level source rocks. Wide range of TOC content variation in our studied samples provides well constraint of organic richness on oil and gas generation and expulsion behaviors and their evolution trajectory during thermal evolution, which will fascinate source rock quality and exploration potential assessment in other source rock systems.

## 1 Introduction

Petroleum expulsion from source rocks is one of the most important but poorly understood subsurface processes. It is generally controlled by temperature history and the original hydrocarbon generation potential of source rocks in a sedimentary basin. The expulsion efficiency increases systematically with an increase in the initial kerogen quality defined by the original hydrogen index (HI_o_) or H/C atomic ratio once the source rock is matured.^1−3^ By reducing the initial total organic carbon (TOC_o_) content of the source rock, the bitumen production in the source rock will reduce dramatically, which in turn leads to a decrease in the expulsion efficiency.^3−5^ However, the expulsion efficiency is also related to the ability of kerogen to retain the generated petroleum, pore size distribution, permeability, and microfracture system in the source rocks.^6,7^ Natural case histories and laboratory experiments are two main ways to investigate petroleum expulsion. The former relies on empirical observations to quantify the differences in product yields between immature and mature source rocks,^3,5,8−10^ while the latter depends on experimental methods (hydrous pyrolysis, closed anhydrous pyrolysis, and open anhydrous pyrolysis) to quantitatively estimate the generation potential and expulsion efficiency of petroleum from source rocks.^11−13^

Rock-Eval is one of the most commonly used anhydrous open pyrolysis systems.^14^ The expulsion efficiency is estimated on the basis of the HI and TOC variation from immature to highly mature samples. As the generated products are removed simultaneously at the time of formation, Rock-Eval pyrolysis obtains the maximum potential of petroleum generation and the highest expulsion efficiency from the source rocks.^5,10,15^ The closed pyrolysis systems, including gold tubes,^16^ microscale sealed vessels,^17^ and various other confined chambers,^18^ can be run under hydrous or anhydrous conditions, but the generated petroleum is not allowed to migrate away from the system and not suitable to simulate expulsion. The semi-open pyrolysis system has some advantages to investigate the expulsion behavior as the expelled oil and gas can be quantitatively characterized.^13,19^ One immature source rock sample has been heated to different target temperatures with a continuous product collection.^13^ However, source residual after heating cannot be collected during the experiments, and no physical change in the source rock can be characterized. Meanwhile, the impact of systematically variable organic richness on expulsion behavior has not been documented in the literature. A comparison of an artificially matured sample series with natural evolution samples may improve the insight into the expulsion process, but various other impacts in nature can hardly be constrained.^1,10,11^ The numerical kinetic models of petroleum expulsion still largely rely on the Rock-Eval pyrolysis experiments, likely causing the overestimation of the expulsion efficiency.^2,20,21^

During the process of thermal maturation, the hydrocarbon generation potential from the source rock declined continuously with increasing levels of thermal maturity due to petroleum generation and expulsion.^22,23^ The Rock-Eval pyrolysis technique defines the current state of the source rock by measuring both the free oil content (*S*1) and the remaining potential (*S*2)^14^ but is hindered by uncertainty regarding the original hydrocarbon-generating potential of the spent organic matter at a high maturity level. The reconstruction of the TOC_o_ and HI_o_ at the immature stage of kerogen is critical for resource appraisal and exploration. Several theoretical and experimental methods have been proposed in the literature to obtain the correlation between the current status and their original values.^24−29^ The evolution trajectory of typical kerogen types on the basis of Rock-Eval pyrolysis or kerogen elemental analysis has typically been applied for TOC loss estimation.^25−29^ However, this approach has an unstated assumption that all generated oil has been expelled from the source rocks, and the TOC reduction is inevitably overestimated. Lewan et al.^24^ proposed two end-member models to determine the TOC_o_ of the thickening mature source rocks: steady-state and dilution. Jarvie et al.^29^ suggested the use of the nearest relevant immature sample as a proxy or statistic value from similar kerogen types for TOC_o_ estimation. Devine^30^ proposed a novel method to predict HI_o_ on a cross plot of *T*_max_–HI with an assumed “vanishing point.” Obviously, these approaches (and their variations) either depend substantially on unreliable assumptions or are currently too subjective for effective evaluation. An appropriate method to estimate the original hydrocarbon generation potential that characterizes the immature kerogen associated with each sample remains uncertain.

During the process of thermal maturation, the hydrocarbon generation potential from the source rock declined continuously with increasing levels of thermal maturity due to petroleum generation and expulsion.^22,23^ The Rock-Eval pyrolysis technique defines the current state of the source rock by measuring both the free oil content (*S*1) and the remaining potential (*S*2)^14^ but is hindered by uncertainty regarding the original hydrocarbon-generating potential of the spent organic matter at a high maturity level. The reconstruction of the TOC_o_ and HI_o_ at the immature stage of kerogen is critical for resource appraisal and exploration. Several theoretical and experimental methods have been proposed in the literature to obtain the correlation between the current status and their original values.^24−29^ The evolution trajectory of typical kerogen types on the basis of Rock-Eval pyrolysis or kerogen elemental analysis has typically been applied for TOC loss estimation.^25−29^ However, this approach has an unstated assumption that all generated oil has been expelled from the source rocks, and the TOC reduction is inevitably overestimated. Lewan et al.^24^ proposed two end-member models to determine the TOC_o_ of the thickening mature source rocks: steady-state and dilution. Jarvie et al.^29^ suggested the use of the nearest relevant immature sample as a proxy or statistic value from similar kerogen types for TOC_o_ estimation. Devine^30^ proposed a novel method to predict HI_o_ on a cross plot of *T*_max_–HI with an assumed “vanishing point.” Obviously, these approaches (and their variations) either depend substantially on unreliable assumptions or are currently too subjective for effective evaluation. An appropriate method to estimate the original hydrocarbon generation potential that characterizes the immature kerogen associated with each sample remains uncertain.

Another controversial issue is the expulsion threshold for oil and gas in the effective source rocks. The expulsion threshold refers to the critical point at which the generated hydrocarbons can be expelled from the source rock to the migration route, while it is still unclear whether the amount of hydrocarbon retained in the source rock needs to meet a certain saturation threshold in the pore space or exceed the respective sorptive capacity of the residual organic carbon or both.^3,11,31−33^ Pepper^3^ assumed that around 200 mg/gC oil is adsorbed onto kerogen surfaces and suggested that source rocks with HI_o_ < 200 mg/gC are unable to expel oil but may expel some gas once thermal cracking of retained oil commenced. The Rock-Eval data set suggested that hydrocarbon expulsion from source rocks is efficient when the original generation potential (*P*_o_ = *S*1_o_ + *S*2_o_) exceeds 5 mg/g, while it is inefficient when *P*_o_ is less than 5 mg/g.^3,5,9^ Once this threshold is reached, the expulsion efficiency will be governed by the pressure gradient and bulk permeability of the source rocks.^11^ There are two methods to predict the lower limit of TOC of effective source rocks. One is based on the original hydrocarbon generation potential and mass balance calculation.^34^ The other is based on the empirical observation of the start of the effective oil window, which corresponds to the initial decrease in the Rock-Eval-derived *S*1/TOC ratio in natural evolution profiles.^35,36^ The difficulty encountered in the previous studies is either caused by limited simulation sample numbers or unconstrained geological heterogeneities.^11,12,37^ The shortcoming of the Rock-Eval data is also sourced from the loss of volatile hydrocarbons before the sampling process,^15,38^ which is difficult to restore. The lower TOC limit for effective oil and gas expulsion has not been determined yet. Meanwhile, systematic experimental data with variable TOC contents at differing temperatures are not available in the literature as well.

To test the effect of organic richness in source rocks on expulsion behavior, a series of immature source rock samples from the same geological unit with different TOC_o_ contents have been simulated in the house-made device, which is a semi-open pyrolysis system with large capacity (kg level) and precise pressure control. A full range of hydrocarbon generation and expulsion has been performed at a slow heating rate to ensure reliable data generation from the experiments. The aim of this work is to establish an evaluation method for oil and gas expulsion from source rocks with variable TOC contents and to determine the lower TOC limit in effective source rocks.

## 2 Samples and Methods

### 2.1 Sample Background

**Figure 1:**
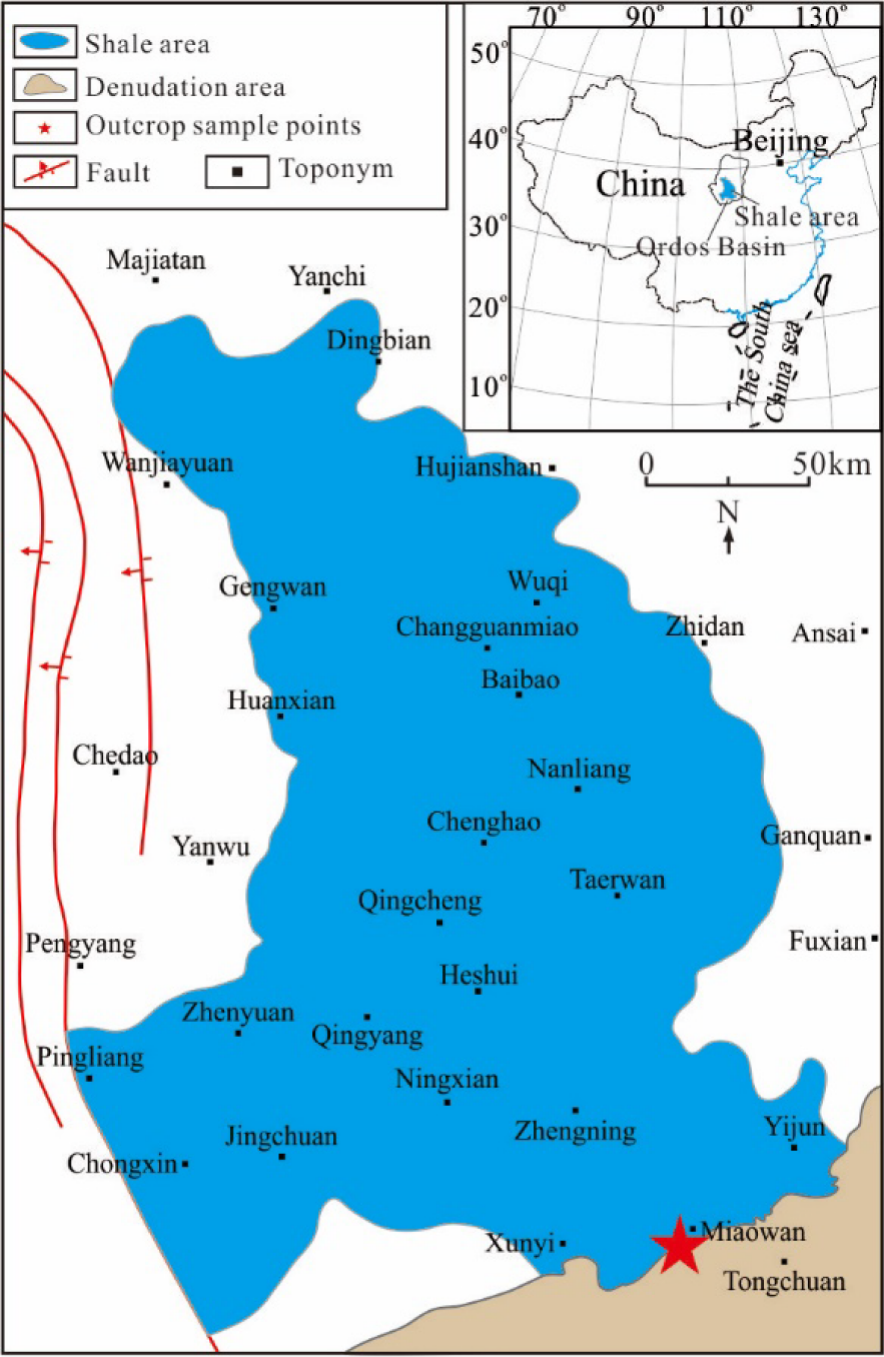
Shale distribution in the Ordos Basin and the sampling sites.

The studied samples were collected from the Triassic Yanchang Formation outcropped at the basin margin of the Ordos Basin, NW China. The Seventh Member of the Yanchang Formation (abbreviated as Chang 7 Member) is a set of black and highly organic enriched lacustrine shale interval. During the deposition of the Chang 7 Member, flourished plankton and anoxic conditions result in the most important source rocks for both conventional and unconventional oil and gas discovered in the basin. The Chang 7 shale covers an area of approximately 5 × 10^4^ km^2^. The TOC contents in the Chang 7 shale vary from 3.4% to 24.4%, and the organic matter is dominated by Type-II kerogen.^39^ The Chang 7 Member source rock samples were obtained from unweathered outcrops excavated at a depth of 5 m in the southeastern margin of the Ordos Basin (Figure 1). Immature samples were collected from nine sampling sites where TOC contents vary considerably to ensure our simulation encountering variable source rock quantities for oil and gas generation and expulsion. All samples have experienced the same geological history and have similar mineral compositions except for a larger amount of silica in the organic-lean ones. Samples collected from each sampling site were crushed into particles of 40–60 mesh and homogenized. The mixed samples were divided into 15 portions, 4 of which were reserved (for supplementary experiments in case of leakage).

### 2.2 Pyrolysis Device

**Figure 2:**
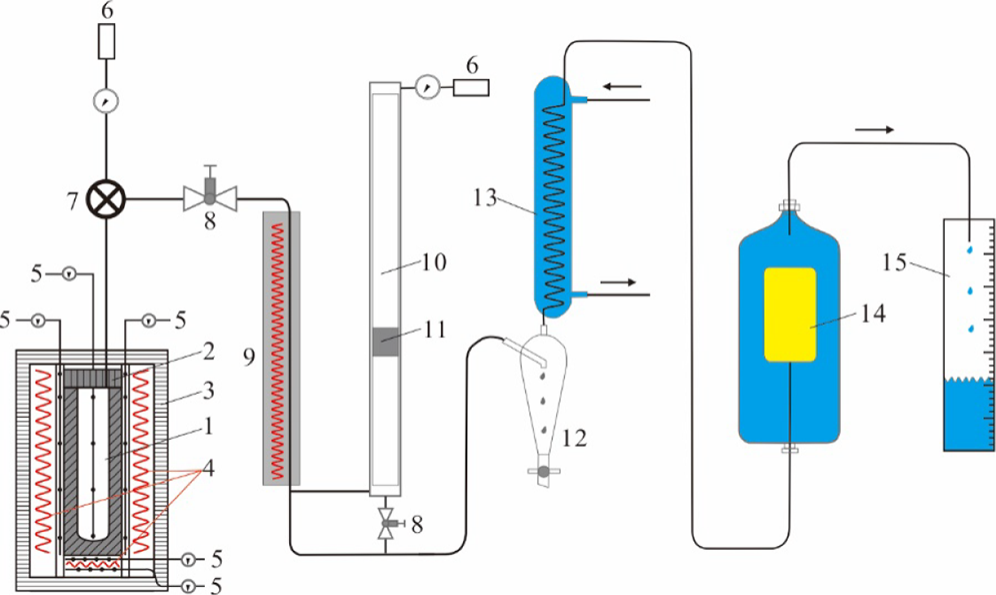
Schematic diagram of the pyrolysis device: (1) autoclave; (2) sealing cover; (3) outer cover; (4) electric heater; (5) thermocouple; (6) pressure gauge; (7) three-way valve controlled by solenoid valve; (8) needle valve; (9) heater; (10) synchronous collector; (11) floating piston; (12) liquid collector; (13) condenser; (14) gas bag; and (15) measuring cylinder.

The simulation device of hydrocarbon generation and expulsion from an immature source rock has been carried out using a house-made instrument shown in Figure 2.^34,40−42^ The reaction container with a volume of 1360 cm^3^ can hold >2 kg specimen. It is made of special alloys that can resist H_2_S, CO_2_, and H_2_ corrosion. The operating temperature can be set in the range of 0–700 °C and the confining pressure is in the range of 0.1–40 MPa. The bottom and lateral heating systems work together to ensure that homogenized heating has been applied. Multiple thermocouples and pressure sensors are equipped to control the temperature and pressure in the reactor. The temperatures were controlled and monitored within a standard error less than ±2 °C, and the pressures were within ±0.2 MPa.

Before the experiment, the homogenized sample was placed in the reactor, which was vacuumed, repeatedly compressed under 20 MPa until the particles were tightly packed. Helium was subsequently refilled in the sample to ensure that air inside the reactor was completely displaced. The system was tested for leakage to ensure that the sample was properly loaded and sealed. During the experiment, the hydrocarbon expulsion was controlled by a solenoid-activated three-way valve. The piston valve was closed initially to seal the reactor. When the generated products continuously accumulated and the pressure of the fluid exceeded the preset pressure of about 5 MPa (fracture pressure caused by hydrocarbon generation in fields), the expulsion valve was opened and the expelled hydrocarbons were bled out automatically. Once the pressure decreased, the solenoid valve was closed again until the next round of pressure builds up. The pipeline for hydrocarbon expulsion was wrapped with a heating belt to prevent the condensation of the generated products. When the products reached the condenser (cooled by 20 °C circulating water), water and liquid hydrocarbons (including C_5_) were accumulated in the collector. Uncondensed gases (C_1_–C_4_, etc.) were collected in a gas bag immersed in a water tank. By the end of the heating process, free oil and gas fluid were collected and quantified. The total moles of the generated gas were calculated using the ideal gas law with the recorded volume, temperature, and pressure at the end of the experiment.

### 2.3 Experimental Design and Temperature Program

The experiments aimed at the expelled oil and gas from source rocks to investigate the quantity and features of hydrocarbon expulsion at different maturity levels. A two-stage heating program was applied in this study. The reactor vessel for the first subsample was heated at a rate of 20 °C/d from ambient temperature to 200 °C and then to 250 °C at a rate of 5 °C/d and isothermally heated for 10 h. For the second subsample, a heating rate of 20 °C/d was applied from room temperature to the previous target temperature (250 °C) and switched to a rate of 5 °C/d to the second target temperature (300 °C) and isothermally heated for 10 h. The same rule was applied for all 11 temperature targets at 250, 300, 320, 335, 350, 360, 390, 440, 500, 540, and 580 °C. The designed heating temperatures cover a full range of petroleum generation from early bitumen generation to maximum gas generation.

To make sure no leakage occurs during and after pyrolysis, a mass balance has been performed for each temperature point on the basis of eq 1
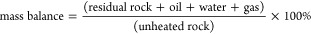
1

If the summed residual and product is <99.5% of raw sample weight, a leakage may occur in this sample and a repeated run has been performed. In our 99 runs, 11 temperature points do not meet the requirement and have been repeated. Therefore, the reliability of all results has been ensured.

### 2.4 Analytical Methods

Rock-Eval pyrolysis analysis was performed on a Vinci Technologies’ Rock-Eval 6 Turbo device following standard procedures.^43^ Aliquots of the original and residual source rocks recovered from the heating experiments were randomly picked from the pulverized sample in an 80 mesh for the analyses. The samples were initially heated at 300 °C for 3 min to obtain the *S*1 peak, representing free volatile hydrocarbons (in mg/g rock), and then heated from 300 to 650 °C at a rate of 25 °C/min to obtain an *S*2 peak, representing the residual hydrocarbon generation potential from organic matter (in mg/g rock). The sum of *S*1 and *S*2 indicates the total hydrocarbon generation potential. The HI is the amount of hydrocarbons (*S*2, mg HC) normalized to TOC in the rock (mg HC/g TOC).^14^ The *T*_peak_ at the maximum yield of *S*2 was converted into *T*_max_ (°C) for the maturity level reference of the source rock.

Vitrinite reflectance measurements were conducted for both raw and heated samples using a Carl Zeiss microscope. The single block was prepared and polished with rock fragments of approximately 2 mm in size embedded in resin. The random vitrinite reflectance (%*R*_o_) of up to 50 particles of vitrinite was measured using an immersion oil method. The analyses were performed at a room temperature of 23 ± 1 °C.

## 3 Results

### 3.1 Geochemistry of Raw Samples

Rock-Eval pyrolysis analysis and vitrinite reflectance (%*R*_o_) measurements were conducted on both raw and heated source rock samples. The TOC_o_ contents vary from 0.51% to 25.99% in nine selected samples and HI_o_ values vary in the range of 388.2–541.5 mgHC/g TOC, showing typical type II kerogen characteristics (Table 1). Uniformly low *T*_max_ values ranging from 427 to 435 °C (with an average value of 430.8 °C) and *R*_o_ of <0.5% indicate an immature nature stage in terms of oil generation (Table 1). The *S*1_o_ values vary from 0.08 to 5.59 mgHC/g rock, and the *S*2_o_ values are in the range of 1.99–138.2 mgHC/g rock. Both of them have a linear correlation with TOC_o_. Although a small amount of free bitumen has been detected especially in organic-rich samples, these samples were still regarded as original samples with an “o” superscript since no organic carbon loss caused by expulsion occurs.

**Table 1:** Basic Geochemical Data of the Original Unheated Source Rock Samples

	no. 1^a^	no. 2	no. 3	no. 4	no. 5	no. 6	no. 7	no. 8	no. 9
TOC_o_ (wt %)	0.51	2.03	3.50	5.03	6.44	8.51	13.34	20.67	25.99
*S*1_o_ (mg/g rock)	0.077	0.317	0.623	0.846	1.305	1.697	2.790	4.482	5.586
*S*2_o_ (mg/g rock)	1.987	8.604	17.283	24.527	32.062	42.422	67.166	111.952	138.198
*T*_max_ (°C)	435	433	429	432	431	433	429	428	427
HI_o_ (mg/g TOC)	388.2	423.0	494.5	487.9	498.2	498.6	503.5	541.5	531.8
*R*_o_ (%)	0.43	0.46	0.47	0.47	0.47	0.47	0.48	0.47	0.48

a Nos. 1–9 represent the nine sampling sites.

### 3.2 Bulk Composition Variation and Yields of Expelled Oil and Gas during the Heating Experiments

**Figure 3:**
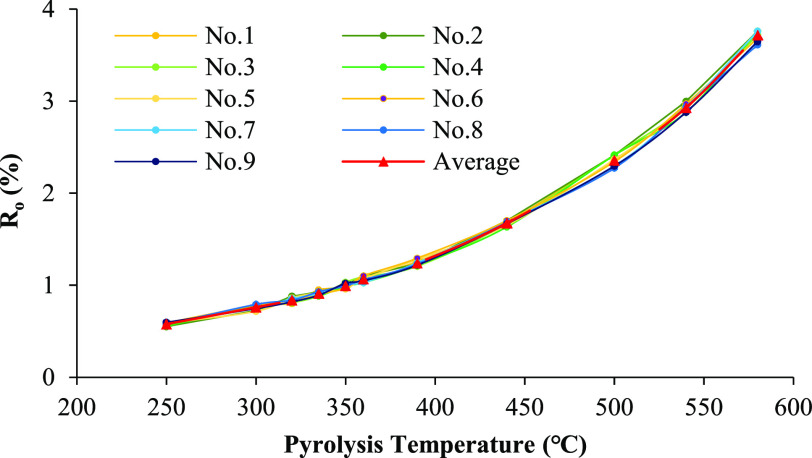
Correlation between pyrolysis temperature and %*R*_o_ in samples from the Chang 7 Member source rocks.

Vitrinite reflectance (*R*_o_) is commonly used to determine the thermal maturity of source rocks and thermal history of petroleum systems while organic matter in source rocks does not always generate petroleum at the same thermal maturity levels.^22,23^ The vitrinite reflectance measurement results for each heated sample are listed in Table 2. While the TOC_o_ contents differ substantially in the studied samples, very similar *R*_o_ values at each heating temperature point can be observed. The *R*_o_ values increase from about 0.47% in raw samples to 3.76% in the highest experimental temperature at 580 °C. The relationship between *R*_o_ values and heating temperatures can be established using eq 2 by using an average *R*_o_ value of nine selected samples at each temperature point (Figure 3)
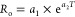
2where *R*_o_ is the vitrinite reflectance (%); *T* is the heating temperature (°C); and *a*_1_ and *a*_2_ are the empirical coefficients equal to 0.13797 and 0.005667, respectively.

**Table 2:** Measured Vitrinite Reflectance (%*R*_o_) Values in Residual Source Rocks after Heating at Different Temperatures

	sample number									
pyrolysis temperature (°C)	no. 1	no. 2	no. 3	no. 4	no. 5	no. 6	no. 7	no. 8	no. 9	average
*R*_o_ (%)										
250	0.57	0.55	0.59	0.56	0.59	0.59	0.58	0.57	0.60	0.58
300	0.78	0.72	0.76	0.78	0.72	0.78	0.75	0.79	0.75	0.76
320	0.80	0.88	0.81	0.84	0.86	0.84	0.85	0.84	0.82	0.84
335	0.95	0.93	0.88	0.88	0.92	0.90	0.91	0.93	0.89	0.91
350	0.98	1.00	1.03	0.99	0.96	0.97	1.01	0.99	1.02	1.00
360	1.09	1.10	1.09	1.03	1.07	1.10	1.03	1.06	1.05	1.07
390	1.26	1.23	1.21	1.22	1.28	1.29	1.24	1.23	1.22	1.24
440	1.68	1.70	1.67	1.63	1.65	1.70	1.68	1.68	1.67	1.67
500	2.41	2.41	2.41	2.41	2.37	2.34	2.30	2.27	2.30	2.36
540	2.88	3.00	2.94	2.88	2.94	2.97	2.94	2.94	2.88	2.93
580	3.76	3.76	3.72	3.76	3.76	3.68	3.76	3.61	3.65	3.72

The TOC content is the most quantitative measurement to evaluate organic richness in source rocks, which decreases systemically with an increase in the heating temperature from the raw sample up to 360 °C, and then remains constant or even gradually increases with further increasing heating temperatures. A more dramatic change occurs in samples with a higher TOC_o_ content. For instance, the leanest organic sample has a TOC_o_ content of 0.51%, which drops to 0.30% once heated at 360 °C, then increases to 0.38 at 500 °C, and remains constant up to 580 °C. The richest organic sample has a TOC_o_ content of 25.99%, which drops to 12.6% once heated at 360 °C but increases to 15.32% at 580 °C (Table 3). A slight increase in the TOC content in highly mature samples is likely caused by the concurrent loss of mineral during heating experiments.

**Table 3:** TOC and HI Values of Residual Source Rock Samples after Heating at Various Temperatures

	sample number								
pyrolysis temperature (°C)	no. 1	no. 2	no. 3	no. 4	no. 5	no. 6	no. 7	no. 8	no. 9
TOC (wt %)									
250	0.51	2.02	3.47	5.02	6.43	8.45	13.25	20.54	25.82
300	0.48	1.87	3.18	4.63	5.85	7.75	12.11	18.92	23.54
320	0.44	1.73	2.96	4.24	5.44	7.21	11.17	17.32	21.27
335	0.37	1.39	2.37	3.44	4.37	5.80	8.99	13.48	16.98
350	0.31	1.18	1.97	2.88	3.68	4.78	7.48	11.51	14.38
360	0.30	1.12	1.76	2.56	3.23	4.45	6.80	10.29	12.60
390	0.33	1.30	2.11	2.97	3.78	5.01	7.64	11.75	14.84
440	0.37	1.37	2.20	3.14	4.00	5.22	8.16	12.12	15.07
500	0.38	1.41	2.25	3.20	4.12	5.38	8.21	12.18	15.17
540	0.38	1.40	2.20	3.29	4.14	5.41	8.22	12.26	15.28
580	0.38	1.39	2.26	3.25	4.16	5.47	8.34	12.29	15.32
HI (mg/g TOC)									
250	386.3	423.9	497.9	487.9	499.6	493.9	496.4	543.5	529.0
300	221.2	245.0	290.8	285.6	283.4	290.5	282.85	313.5	298.9
320	178.8	189.8	216.8	222.3	228.5	224.0	222.3	243.8	235.5
335	139.6	152.1	170.7	169.8	172.5	177.1	177.6	186.9	182.6
350	104.6	115.7	133.0	131.1	138.0	137.19	134.1	149.2	147.0
360	89.8	97.4	115.1	115.6	115.3	114.1	113.4	124.8	122.9
390	61.4	66.1	76.5	76.0	78.1	79.8	77.5	85.9	83.5
440	11.2	12.3	14.0	14.4	14.1	14.0	14.5	15.4	15.1
500	1.4	1.6	1.8	1.8	1.8	1.9	1.9	2.0	2.0
540	0.0	0.0	0.0	0.0	0.0	0.0	0.0	0.0	0.0
580	0.0	0.0	0.0	0.0	0.0	0.0	0.0	0.0	0.0

The HI values fall slightly from an initial status to a heating temperature of 250 °C but decrease drastically with an increase in the heating temperatures. More than 50% of hydrocarbon generation potential has been conversed at 320 °C and is nearly exhausted at 440 °C, where HI narrows to ∼15 mg HC/g TOC or less. No reliable HI value can be obtained from heating temperatures higher than 440 °C (Table 3).

**Figure 4:**
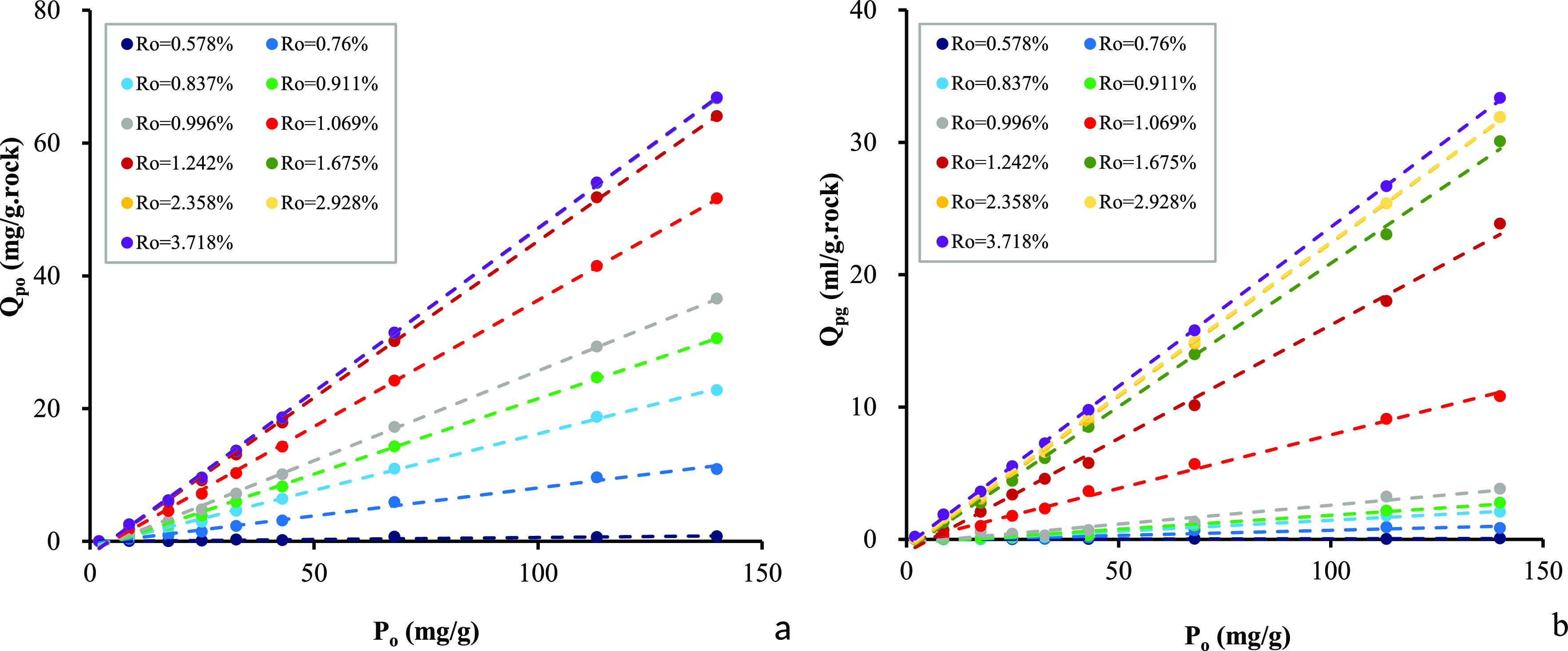
Relationship among cumulative amount of expelled oil (a), cumulative volume of expelled gas (b), and original hydrocarbon generation potential (*P*_o_) at different maturity levels in samples from the Chang 7 Member.

During the heating experiments, oil and gas released from the reactor were collected after the samples were heated at designed temperatures. The yields of cumulative expelled oil (*Q*_po_, in mg/g) and expelled gas (*Q*_pg_, in mL/g) during the pyrolysis are shown in Table 4. The amount of expelled oil and volume of expelled gas increase systematically with an increase in the original total hydrocarbon generation potentials (*P*_o_ = *S*1_o_ + *S*2_o_) and maturity levels (Figure 4).

**Table 4:** Expelled Oil and Gas of the Pyrolyzed Samples with Increasing Pyrolysis Temperatures in Samples from the Chang 7 Member

	sample number								
pyrolysis temperature (°C)	no. 1	no. 2	no. 3	no. 4	no. 5	no. 6	no. 7	no. 8	no. 9
*Q*_po_ (mg/g)^a^									
250	0.00	0.07	0.02	0.11	0.28	0.16	0.70	0.62	0.76
300	0.00	0.37	0.90	1.45	2.31	3.14	5.90	9.63	10.89
320	0.01	0.74	1.94	2.95	4.61	6.39	10.94	18.76	22.78
335	0.01	0.98	2.49	3.86	5.94	8.27	14.29	24.70	30.57
350	0.01	1.23	3.04	4.83	7.19	10.09	17.22	29.33	36.57
360	0.02	1.86	4.56	7.15	10.27	14.28	24.21	41.47	51.66
390	0.03	2.44	5.93	9.19	13.07	17.89	30.15	51.82	64.03
440	0.03	2.55	6.18	9.58	13.61	18.63	31.38	53.93	66.71
500	0.03	2.55	6.20	9.60	13.64	18.67	31.45	54.05	66.85
540	0.03	2.55	6.20	9.60	13.64	18.67	31.45	54.05	66.85
580	0.03	2.55	6.20	9.60	13.64	18.67	31.45	54.05	66.85
*Q*_pg_ (mL/g)^b^									
250	0.00	0.00	0.00	0.00	0.03	0.02	0.03	0.02	0.06
300	0.00	0.00	0.00	0.05	0.03	0.13	0.68	0.92	0.85
320	0.00	0.02	0.00	0.11	0.17	0.28	1.05	1.78	2.07
335	0.00	0.05	0.09	0.19	0.27	0.30	1.08	2.16	2.77
350	0.00	0.08	0.57	0.42	0.28	0.69	1.28	3.22	3.82
360	0.01	0.34	1.00	1.76	2.31	3.63	5.69	9.10	10.81
390	0.02	0.70	2.07	3.38	4.56	5.76	10.12	18.00	23.86
440	0.03	1.26	2.75	4.41	6.13	8.48	13.98	23.04	30.09
500	0.07	1.58	3.07	4.98	6.58	8.86	14.80	25.38	31.90
540	0.07	1.76	3.35	5.32	6.60	9.27	15.31	26.08	32.61
580	0.08	1.88	3.59	5.52	6.60	9.76	15.76	26.67	33.30

a *Q*_po_ refers to the cumulative amount of expelled oil.

b *Q*_pg_ refers to the cumulative volume of expelled gas.

### 3.3 Correlation of Expelled Oil with Organic Content and Maturity Level

**Figure 5:**
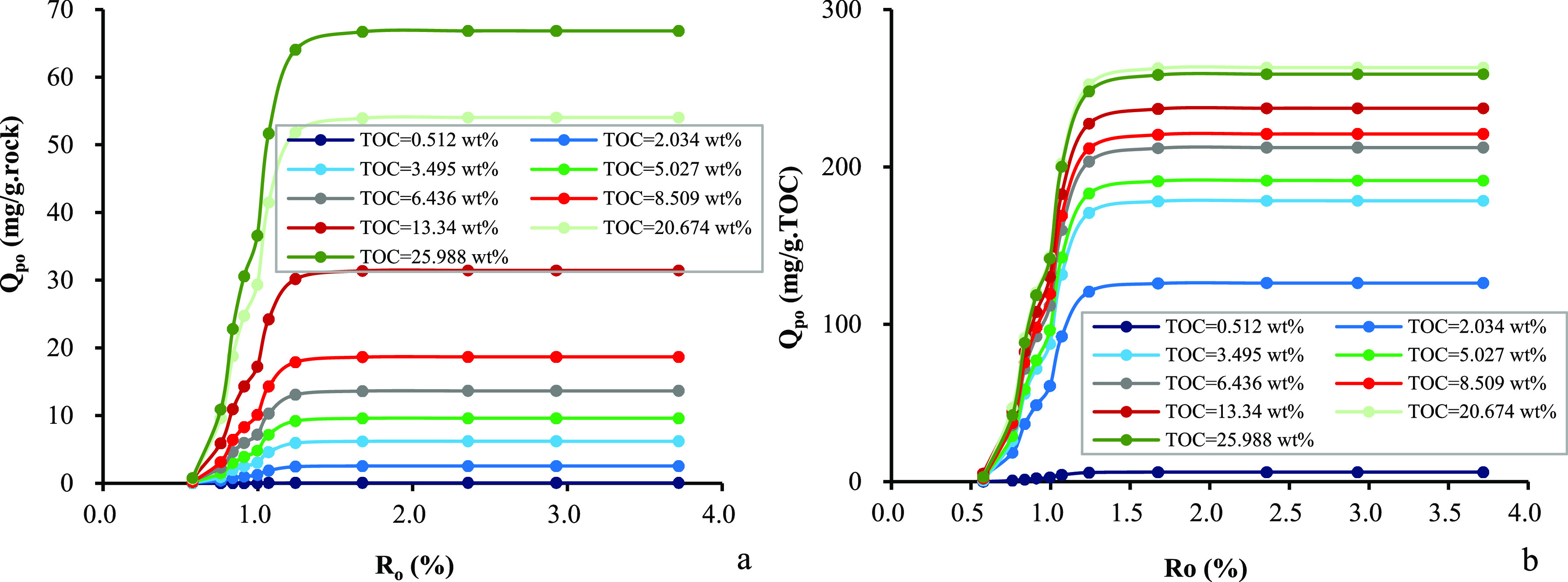
Relationship between *R*_o_ and the cumulative amount of expelled oil in samples from the Chang 7 Member: (a) per unit weight of source rocks and (b) per unit weight of TOC.

The amount of expelled oil from source rock is closely related to *R*_o_ and the TOC_o_ content. Our simulation results illustrated that significant generation begins at *R*_o_ ∼ 0.6%, and the cumulative amount of expelled oil increases sharply with an increase in the maturity level when the *R*_o_ value is <1.25%, slows down significantly in the *R*_o_ range of 1.25–1.45%, and ceases when the *R*_o_ value is >1.65%. The plot of cumulative amounts of expelled oil vs *R*_o_ values shows a very similar pattern but a different magnitude in terms of per unit weight of source rock from per unit weight of the TOC_o_ content (Figure 5).

**Figure 6:**
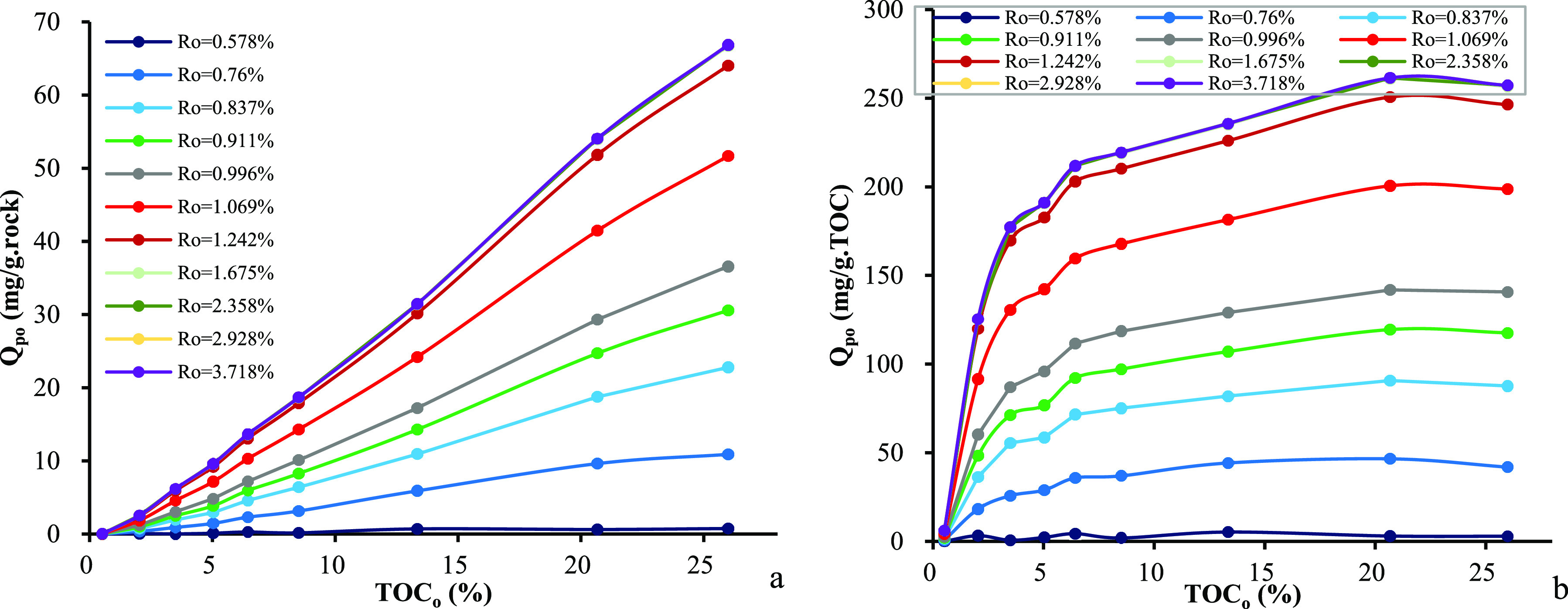
Relationship between the original TOC_o_ content and cumulative amount of expelled oil in samples from the Chang 7 Member: (a) per unit weight of source rocks and (b) per unit weight of TOC.

**Figure 7:**
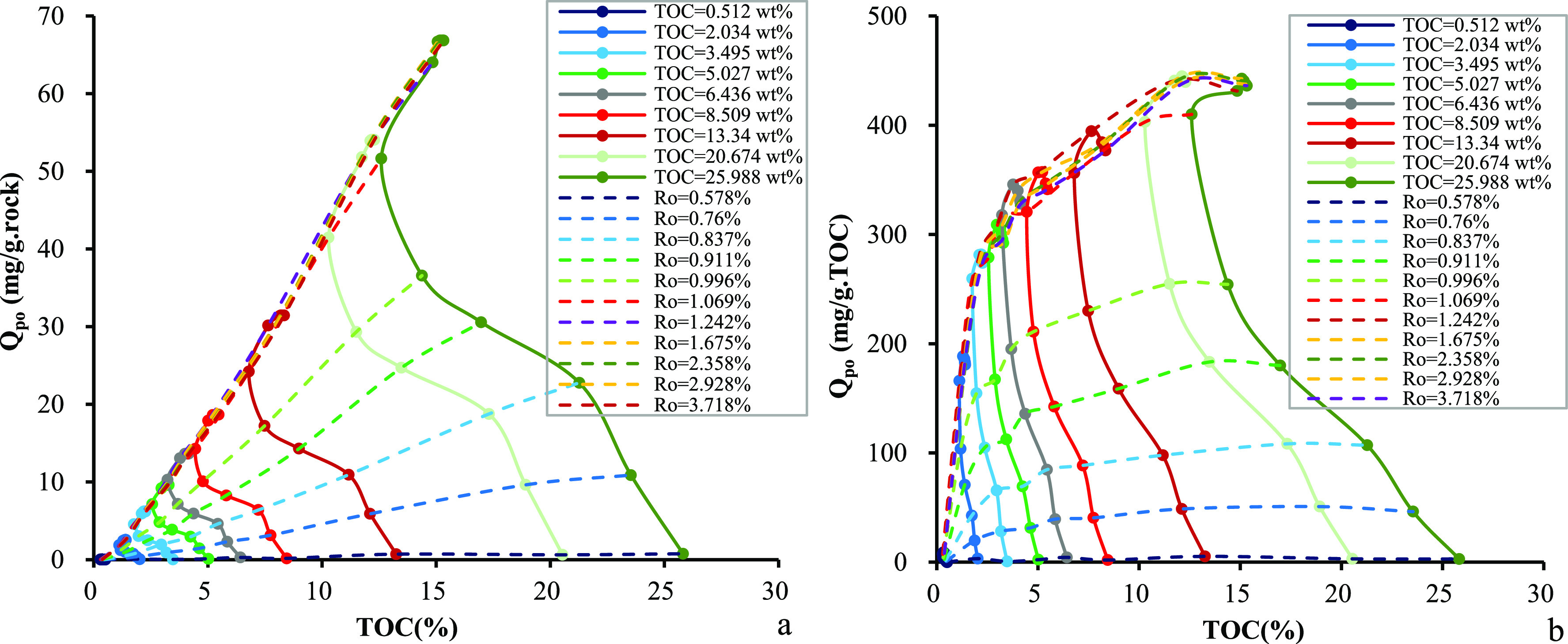
Relationship among TOC content, *R*_o_, and cumulative amount of expelled oil in samples from the Chang 7 Member: (a) per unit weight of source rocks and (b) per unit weight of TOC.

The overall effect of organic richness on the cumulative amount of expelled oil is plotted in Figure 6. The richer the source rock, the larger the amounts of expelled oil that occurs in per unit weight of the source rock (Figure 6a). If per unit weight of TOC is accounted, the relationship between the TOC_o_ content and the amounts of expelled oil shows a different pattern (Figure 6b). A sharp increase in the amounts of expelled oil occurs when the TOC_o_ content increases from 0.5% to 5.0%, and a gentle increase occurs in the TOC_o_ range of 5.0–20.0%, but a slightl decrease occurs when the TOC_o_ content increases further. Figure 7 shows the dependence of the cumulative amounts of expelled oil on *R*_o_ values and TOC contents. The cumulative amounts of expelled oil per unit weight of source rocks increase with the TOC content at any maturity level but the change is less significant when the *R*_o_ value reaches 1.25% (Figure 7a). Approximately, half of the TOC_o_ content has been depleted at the end of the oil window, corresponding to a sharp increase in the cumulative amount of expelled oil per unit weight of TOC. However, the trend is reversed when the *R*_o_ value is >1.25%, partially owing to thermal cracking of retained oil and coke formation (Figure 7b). Numerical correlation among the amount of expelled oil, *R*_o_, and TOC content can be constructed.

### 3.4 Correlation of Expelled Gas with Organic Content and Maturity Level

**Figure 8:**
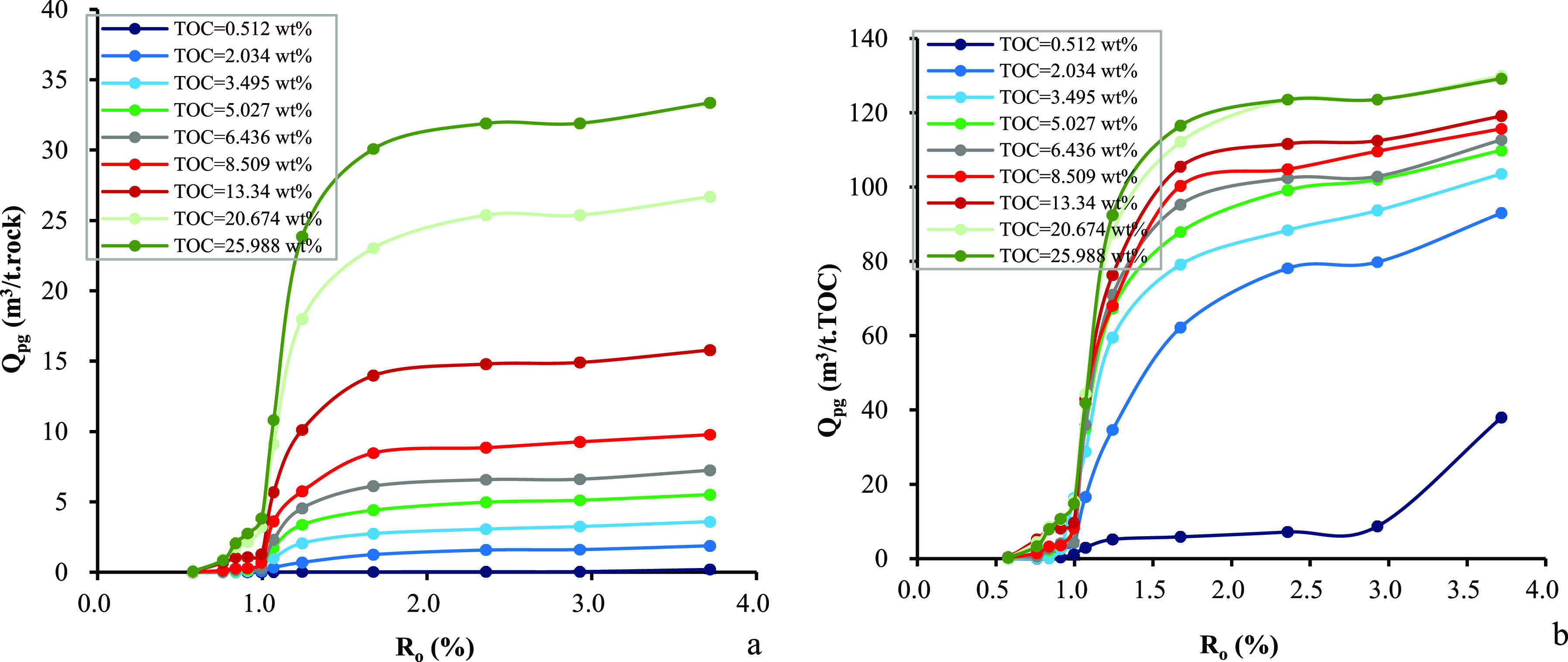
Relationship between *R*_o_ and cumulative volume of expelled gas in samples from the Chang 7 Member: (a) per unit weight of source rocks and (b) per unit weight of TOC.

**Figure 9:**
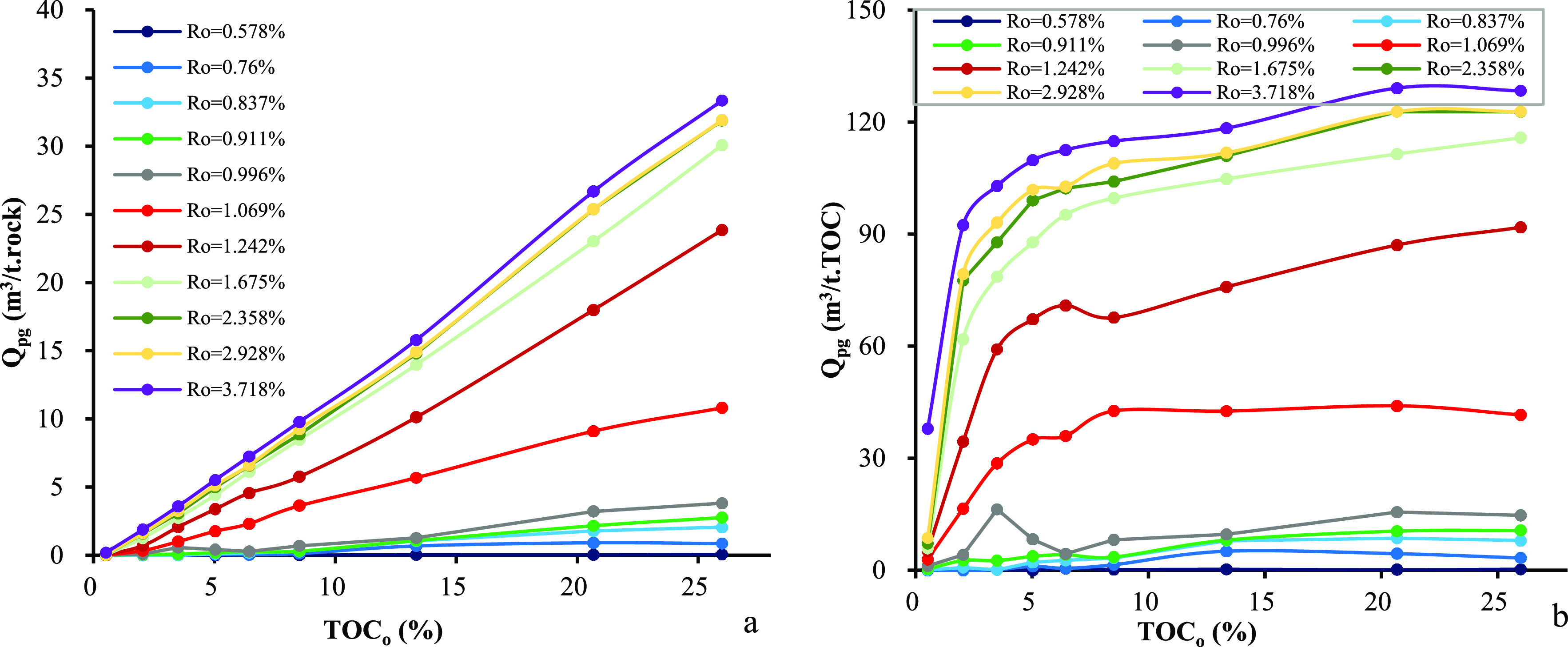
Relationship between the original TOC_o_ content and cumulative volume of expelled gas in samples from the Chang 7 Member: (a) per unit weight of source rocks and (b) per unit weight of TOC.

**Figure 10:**
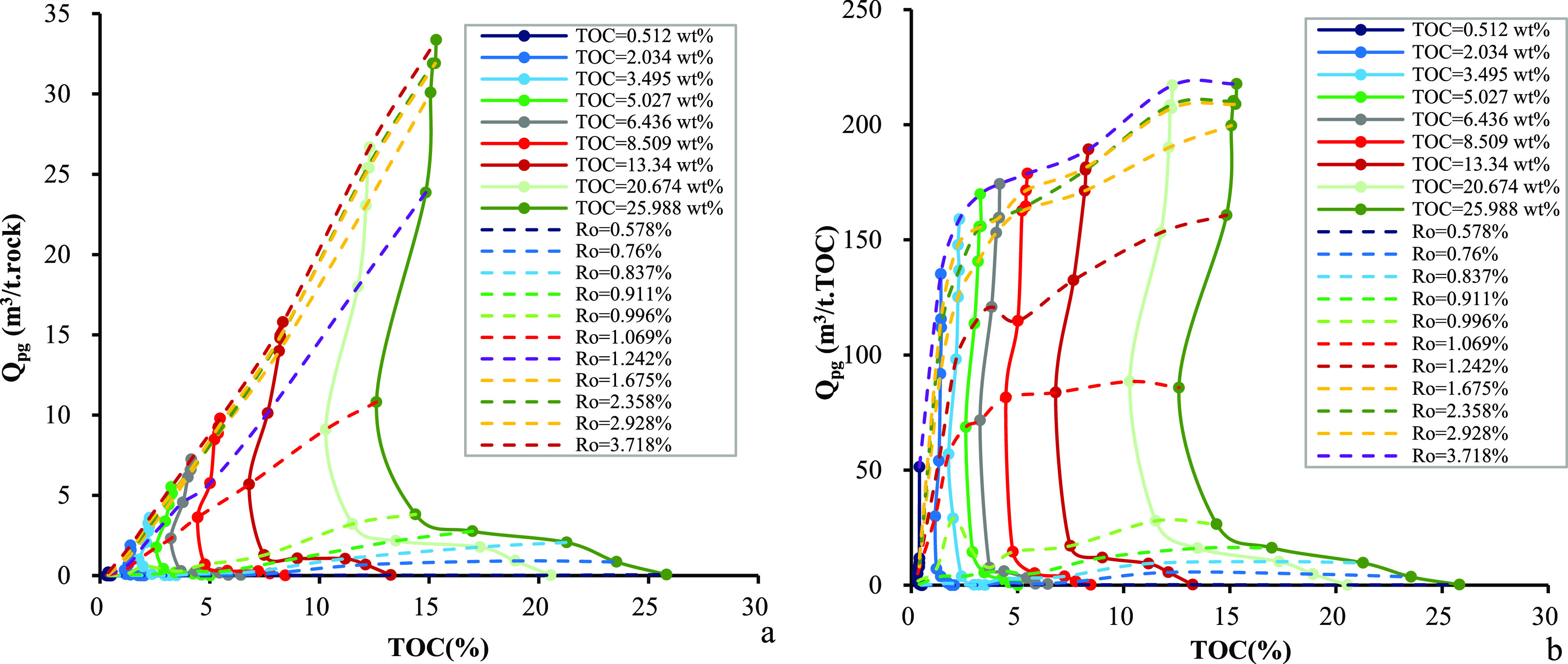
Relationship among TOC content, *R*_o_, and the cumulative volume of expelled gas in samples from the Chang 7 Member: (a) per unit weight of source rocks and (b) per unit weight of TOC.

Similar to the expelled oil, the expelled volume of gas from source rocks is also controlled by the *R*_o_ values and TOC_o_ contents, but the trends are different. The cumulative expelled volume of gas of per unit weight of source rocks is insignificant when the *R*_o_ value is <1.0%. It increases quickly when the *R*_o_ values are in the range of 1.0–2.3% but increases slowly when the *R*_o_ values are >2.3% (Figure 8a). A similar trend can be observed in per unit weight of TOC_o_ in original source rocks except for the organic leanest sample (Figure 8b). The cumulative volume of expelled gas per unit weight of source rocks increases systematically with the TOC_o_ content (Figure 9a). Very low cumulative volume of gas has been expelled when the *R*_o_ value is <1.0%, but it increases drastically in the *R*_o_ values of 1.0–2.3%. The cumulative volume of expelled gas per unit weight of TOC varies with the TOC content in a similar way to the cumulative amount of expelled oil. A dramatic increment occurs in the TOC_o_ range of 0.5–5.0% but a mild increment is observed in higher TOC_o_ contents (Figure 9b). Figure 10 shows the dependence of the cumulative volume of expelled gas on the *R*_o_ values and TOC_o_ contents. The cumulative volume of expelled gas increases with the TOC_o_ content at any maturity level, but the magnitude varies considerably in per unit weight of source rocks from per unit weight of TOC. By increasing the TOC_o_ content to 5% and higher, the cumulative volume of expelled gas from source rocks in per unit of TOC increases slightly, which in turn leads to a platform of expulsion efficiency. Numerical correlation among the volume of expelled gas, *R*_o_ values, and TOC contents has also been established.

## 4 Discussion

### 4.1 TOC Content Variation with Maturity Levels

During the process of thermal maturation, the hydrocarbon generation potential of the source rock has been converted to oil and gas contents under the stress of time and temperature. Determining the TOC_o_ of a source rock is important for total volume of hydrocarbon generation in a perspective area. The TOC reduction during thermal maturation can easily be restored to their initial values by using Rock-Eval pyrolysis and simple calculations for the assumed kerogen types.^25,27,30^ Theoretical estimates of organic carbon reduction from kerogen H/C atomic ratios derive a similar carbon loss estimation.^26^ Another approach is to use average TOC in samples from the same strata at the basin margin with a similar organic type as a surrogate for highly mature samples, and the difference between them was regarded as the loss of TOC during maturation.^28,29^ This study has no intention to further explore the reliability of these assumptions as it has been commented by Devine.^30^ Generally, the maximum loss in organic carbon depends on the kerogen type and the transformation ratio from kerogen to hydrocarbons, which increases continuously with levels of maturity. The estimated TOC reduction for typical of type I, II, and III kerogen are about 70%, 50%, and 20%, respectively, when vitrinite reflectance is about 2.0%.^25^ The difference between these kerogen types is attributed to the initial percentage of dead carbon or inert carbon, which bears no potential for hydrocarbon generation.^5,12,29^

**Figure 11:**
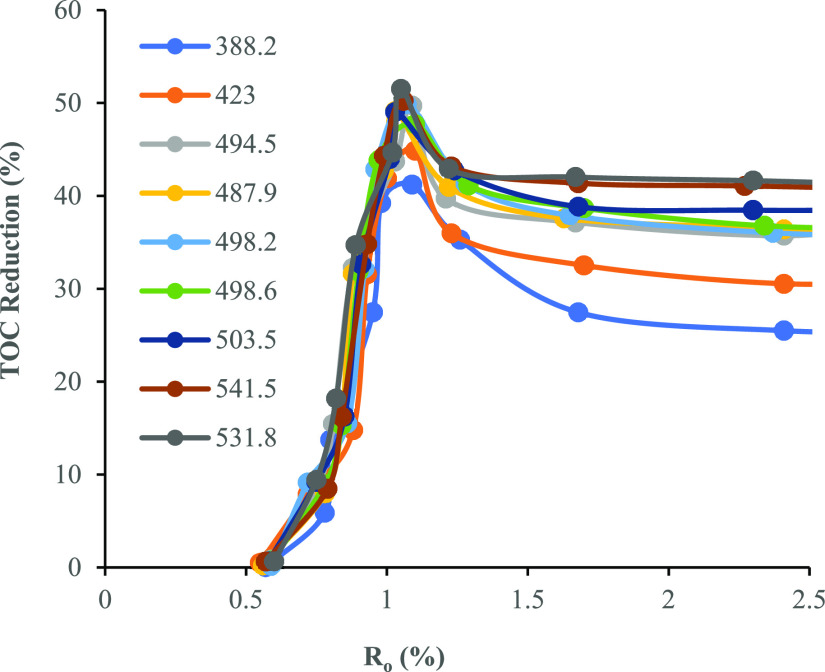
Carbon-loss curves during semi-open pyrolysis experimental results from the Chang 7 Member.

Our heating experimental data present here show a very different evolution trajectory from Rock-Eval based results (Figure 11). The ultimate TOC loss at the highest maturity level varies from 25.5% to 41.1%, which is positively correlated to the TOC_o_ and linearly correlated to HI_o_. The higher the hydrocarbon generation potential, the more the carbon reduction that occurs during maturation. However, the proportion of carbon loss in our results is less than the typical value of 50% for type II kerogens estimated by Daly and Edman.^25^ This is likely caused by different expulsion efficiencies between the two pyrolysis methods. Rock-Eval pyrolysis expels all products out once formed and no secondary cracking of oil occurs, while semi-open pyrolysis in this study retains part of oil, which will further crack to form gas and coke. The tangible difference between the data in our study and those published in the literature is the trend of carbon loss during maturation. Surprisingly, all samples show a rapid carbon loss in the *R*_o_ range of 0.5% to 1.1%, and then the trend reversed in the higher maturity range. The most TOC loss corresponds to the end of oil generation window rather than continuous reduction with increasing levels of maturity. The increase in the TOC contents with increasing levels of maturity has commonly been noticed from coal thermal evolution^35,44^ but has not been reported in the shales, as far as the authors are aware. The increase in the TOC content of coal is attributed to the loss of moisture and various oxygen functional groups through the diagenesis and catagenesis processes.^35^ Vu et al.^44^ reported a series of the Cretaceous–Cenozoic coals from New Zealand with an increase in TOC in the *R*_o_ range of 0.23–0.81%, which falls in the early oil generation window. However, the change in shales may have different mechanisms from coal. Our data indicated that TOC increment only occurs after peak oil generation with *R*_o_ > 1.1%, which is in the late catagenesis and metamorphosis stages. The loss of structural water or bound water in various clay minerals during this stage of evolution is likely the main drive for mineral change and matrix loss, resulting in relatively concentrated organic fractions. For instance, the smectite-to-illite transformation releases large amounts of bound water, which not only accelerates the expulsion process but also reduces the weight of the matrix.^45^ There seems to be a volume/weight issue that needs to be solved, but a full mass balance calculation of organic and inorganic interactions is out the scope of this study. Further investigation is still called for.

### 4.2 Original Generation Potential (P_o_) Evaluation

The most important characteristic to assess an active source rock deposit is to define its initial organic matter quantity (TOC_o_) and the original kerogen quality (HI_o_) when the source rock is at the immature stage. Similar to the TOC_o_ reconstruction, the change in HI from the initial status to the present-day value has been widely discussed in the literature.^12,21,28,29^ The Rock-Eval pyrolysis technique has largely been applied for such purpose. However, the current state of the retained hydrocarbon (*S*1) and the remaining potential (*S*2) are quite different from their original potential (*S*2_o_) due to oil and gas expulsion. The empirical methods to assess the original oil and gas potentials in source rock horizons rely upon mapping the Rock-Eval *S*2 and TOC in the study area with a wide maturity range and build forward modeling protocol.^21,28^ Alternatively, Rock-Eval *S*2 vs TOC plot of different maturity samples with known kerogen types was used to build the evolution temperate, and different slopes were used to extrapolate the HI_o_ and TOC_o_. The original hydrocarbon generation potential at different thermal maturity levels can be restored using assumed organic matter types. For instance, the average HI_o_ value for the Barnett Shale was calculated based on 95% of type II kerogen and 5% of type III kerogen at the transformation ratio of 0.95.^29^ Devine^30^ proposed a novel method to predict the original HI on a cross plot of *T*_max_ (linear scale) vs HI (log 10 scale) from high-*T*_max_, low-HI “vanishing point” to an “immature horizon” to extrapolate the HI_o_ value. There is no doubt for practical application of those approaches; however, the uncertainty remains high due to heterogeneity of source rocks in any petroleum systems. Generally, it is difficult to accurately re-establish the original hydrocarbon generation potential for source rocks when *R*_o_ is >1.2% due to the depletion of the hydrocarbon generation potential.

The data present here have some advantages to cover the widest range of TOC_o_ contents in an immature stage and a complete oil and gas generation and expulsion processes in lacustrine type II kerogen. The original hydrocarbon generation potential in this study is defined as the product of TOC_o_ and HI_o_, which is the same as *S*2_o_ when no hydrocarbon has been generated in the source rock itself (*S*1_o_ = zero). While small amount of *S*1 has been detected in our raw samples, the sum of *S*1 and *S*2 in raw samples should remain at or near the original *S*2_o_ as no expulsion occurs. Once the effective expulsion occurs during the heating experiments, *S*1 + *S*2 is lower than *S*2_o_. The measured hydrocarbon generation potential (*P* = *S*1+ *S*2) has progressively declined from the near original potential (*P*_o_) at 250 °C to zero at 540 °C.

The current *P* at any maturity stage can be linked back to *P*_o_ via *R*_o_ (eq 3)
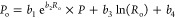
3where *P*_o_ is the original hydrocarbon generation potential of the source rock samples to be tested (mg/g); *P* is the hydrocarbon generation potential corresponding to certain *R*_o_ of source rocks to be evaluated (mg/g TOC); and *b*_1_, *b*_2_, *b*_3_, and *b*_4_ are the empirical coefficients equal to 0.0668, 4.5715, −3.9872, and 2.396, respectively.

Such calculation is well constrained without uncertainty and allows wide maturity range for original hydrocarbon generation potential estimation, while the correlation of other kerogens may behave differently.

### 4.3 Lower TOC Limit for Effective Oil and Gas Source Rocks

Oil expulsion and primary migration will not start until a sufficient amount of oil is produced within the source rocks.^3,46^ The effective source rock can expel a large quantity of oil from a source rock, while the ineffective source rock retains most of the generated oil within the source rock. The expulsion threshold is defined as the critical point at which the source rocks have generated enough oil to be able to expel from source rocks.^47^ Previous experiments have illustrated that the amounts of expelled oil declined significantly from organic-rich source rocks to organic-lean source rocks, and the richness of the source rock is the primary control on the expulsion efficiency of a source rock.^11^ However, efficient expulsion seems to be a function of various parameters such as the distribution of the source potential, adsorption ability of kerogen, pore size distribution of the source rock, and development of the microfracture system. Some studies suggested a certain saturation threshold by default, which means certain percentage of the pore volume must be filled with oil before expulsion will occur. If the saturation threshold increases, the hydrocarbon expulsion will be delayed.^48^ Therefore, the minimum requirement of the TOC content for effective expulsion is difficult to quantify.

The correlation between the cumulative amount of expelled oil and initial organic matter quality at any maturity level has been established by our heating experiments. A wide range of TOC shows a very similar expulsion behavior except for the leanest organic content sample as they share a common organic matter type. The lower limit of TOC for effective source rocks can be established from the relationship between the *R*_o_-related TOC content and cumulative quantity of the expelled oil during the pyrolysis experiments (eq 4)
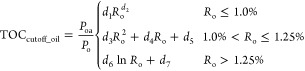
4where TOC_cutoff_oil_ is the lower limit of TOC for effective source rocks (%); *R*_o_ is the vitrinite reflectance (%); and *d*_1_, *d*_2_, *d*_3_, *d*_4_, *d*_5_, *d*_6_, and *d*_7_ are the empirical coefficients equal to 0.6184, −2.2080, 11.1853, −25.0319, 14.4632, 0.0264, and 0.6196, respectively.

**Figure 12:**
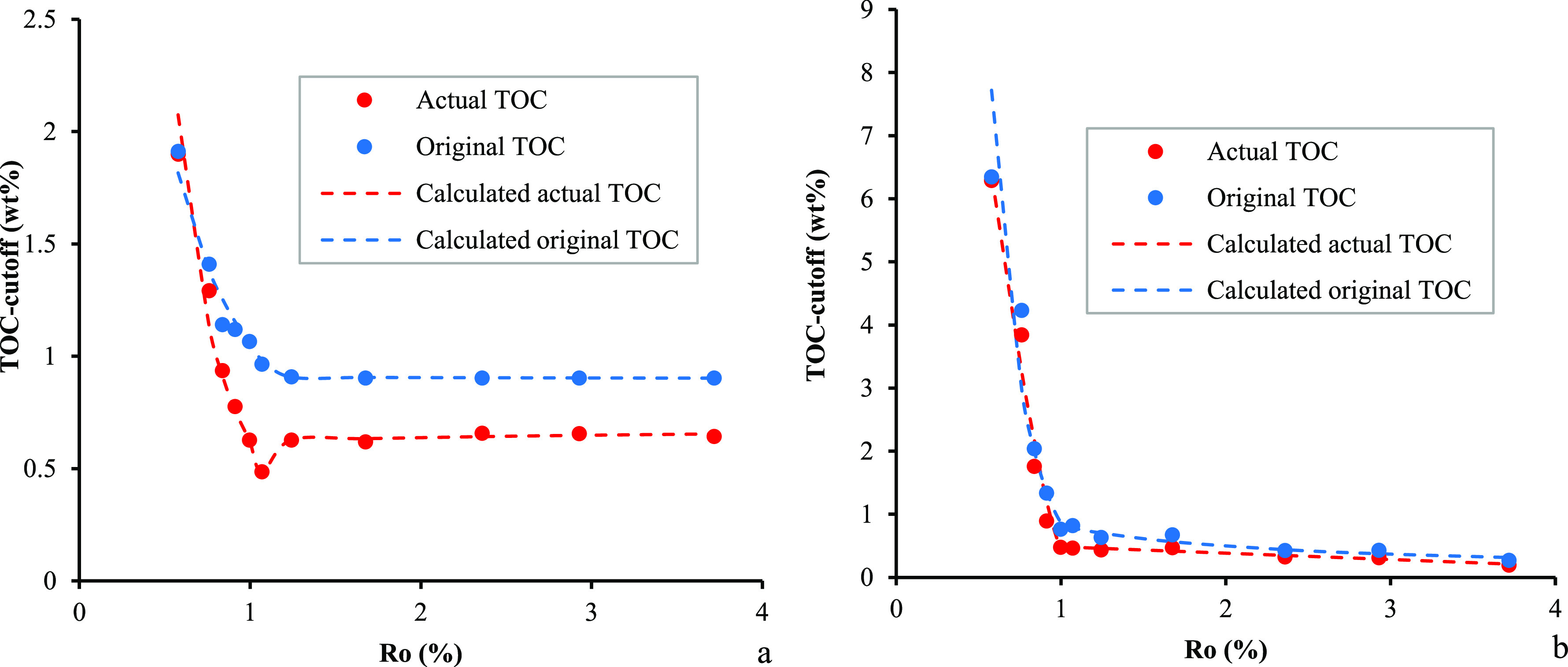
Comparison between the calculated and measured values for the lower limit of TOC in effective source rocks in samples from the Chang 7 Member. (a) Effective oil source rock and (b) effective gas source rock.

Thus, the lower limit of the TOC content can be inferred from the established correlation between the amount of expelled oil and the TOC content at any maturity level. The lower limit of the TOC = 0.5 wt % occurs at about 1.05%*R*_o_ and a higher maturity range in our studied samples, corresponding to the lower limit of the original TOC_o_ content of 0.91 wt % (Figure 12). This means that the TOC_o_ of 0.91 at the immature stage for type II kerogen is required for sufficient amounts of the oil to saturate the pore space and/or exceed the adsorption ability when the source rock reaches the peak oil generation stage. A much higher TOC value is required at an early oil generation stage. The highest lower limit of 2.0 wt % TOC at the earliest oil generation stage suggests that if the initial TOC content is higher than 2.0 wt %, the expulsion will be very efficient. The minimum TOC line, thus, corresponds to the maturity range within which the start of the effective oil window generally occurs for the lacustrine type II kerogen data set.

Similarly, the lower limit of TOC for effective gas source rocks can be obtained by the relationship between the *R*_o_-related TOC content and the cumulative volume of expelled gas (eq 5)
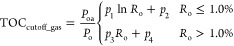
5where TOC_cutoff_gas_ is the lower limit of TOC for gas accumulation (%); *R*_o_ is the vitrinite reflectance (%); and *p*_1_, *p*_2_, *p*_3_, and *p*_4_ are the empirical coefficients equal to −11.3558, 0.1605, −0.1005, and 0.5844, respectively.

Our model predicts that the lower limit of TOC is 0.48 wt % at *R*_o_ of 1.0%, corresponding to the original TOC_o_ content of 0.76%. The lower limit of TOC is 0.2% when the *R*_o_ value reaches 3.5%. However, the lower limit of TOC for effective gas source rocks increases sharply with decreasing maturity levels, suggesting that no gas expulsion should take place until a high maturity level is reached. While the evaluation model is base the lacustrine type II kerogens and the result depends largely on the experimental conditions, the recognition of maturity related to lower TOC limit for effective gas source rocks provides an analogue for all petroleum systems.

## 5 Conclusions

Semi-open pyrolysis experiments have been performed on a suite of outcrop source rock samples with variable TOC_o_ contents from the Triassic Chang 7 Member, Yanchang Formation in the Ordos Basin, NW China. Two-stage slow heating rates in a high-capacity reactor enable the expulsion of substantial amounts of oil and volume of gas for product quantification and residual characterization. While the initial organic richness varies considerably, the measured *R*_o_ values at each heating temperature are quite uniform and their relationship can be easily established. The TOC contents decrease markedly during heating experiments when *R*_o_ is <1.1% but increases slightly or remain constant with a further increase in the maturity level. The relationship among the amount of expelled oil, volume of expelled gas, *R*_o_ values, and TOC contents can be numerically constructed. After the threshold of oil generation (*R*_o_ of 0.6%), the cumulative expelled oil increased sharply when *R*_o_ is <1.25%, but oil expulsion is terminated when *R*_o_ is >1.45%. The cumulative amount of expelled oil and cumulative volume of expelled gas increase linearly with the TOC_o_ content per unit weight of source rocks; however, a dramatic increase only occurs in the TOC_o_ range of 0.5–5.0%, a mild increase in the TOC_o_ occurs in the range of 5.0–20.0%, and a slight decrease occurs in the TOC_o_ of >20.0% in terms of per unit weight of TOC. The lower limit of TOC in matured source rock and its corresponding initial value for effective oil and gas expulsion has been inferred on the basis of established numerical correlations. The minimum TOC of 0.5% is required for effective oil expulsion from source rocks at the *R*_o_ value of 1.05% or higher, corresponding to the TOC_o_ of 0.91% at its immature stage. The lower limit of TOC is 0.48 at an *R*_o_ value of 1.0% and is 0.3% at an *R*_o_ value of 3.5% for effective gas expulsion, corresponding to the TOC_o_ of 0.76%. However, the lower limit TOC cutoff of an effective source rock is much higher when the maturity level is lower than the above-mentioned value. Our experimental results provide deep insight into oil and gas expulsion processes during thermal evolution and a practical tool for the original organic matter content and hydrocarbon generation potential restoration and minimal TOC cutoff estimation for effective oil and gas source rocks in lacustrine type II kerogen, while other kerogen types may behave differently.
